# Danger Signals Activating the Immune Response after Trauma

**DOI:** 10.1155/2012/315941

**Published:** 2012-06-19

**Authors:** Stefanie Hirsiger, Hans-Peter Simmen, Clément M. L. Werner, Guido A. Wanner, Daniel Rittirsch

**Affiliations:** Division of Trauma Surgery, Department of Surgery, University Hospital Zurich, 8091 Zurich, Switzerland

## Abstract

Sterile injury can cause a systemic inflammatory response syndrome (SIRS) that resembles the host response during sepsis. The inflammatory response following trauma comprises various systems of the human body which are cross-linked with each other within a highly complex network of inflammation. Endogenous danger signals (danger-associated molecular patterns; DAMPs; alarmins) as well as exogenous pathogen-associated molecular patterns (PAMPs) play a crucial role in the initiation of the immune response. With popularization of the “danger theory,” numerous DAMPs and PAMPs and their corresponding pathogen-recognition receptors have been identified. In this paper, we highlight the role of the DAMPs high-mobility group box protein 1 (HMGB1), interleukin-1**α** (IL-1**α**), and interleukin-33 (IL-33) as unique dual-function mediators as well as mitochondrial danger signals released upon cellular trauma and necrosis.

## 1. Introduction

Trauma and tissue damage trigger an inflammatory response, which is required for postinjury regeneration and tissue repair. In the case of severe trauma, an overwhelming, systemic inflammatory response can result in additional multi-organ damage to the host cells and the development of multi-organ failure (MOF) [1]. The inflammatory response after severe trauma correlates with the severity of injury and is associated with mortality and the development of complications, such as MOF or sepsis [2, 3]. Inflammation following tissue damage is a dynamic process, which is driven by numerous inflammatory mediators. The innate and the adaptive immune system can be activated by endogenous signals that originate from stressed, injured, or necrotic cells, signifying “danger” to the host [4]. The notion that endogenous and exogenous molecular patterns can cause a similar host response through the same receptors challenged the model of the immune system discriminating between “self” and “nonself”, but could be sufficiently explained by the “danger theory.” In 1994, the “danger theory” of the inflammatory response following trauma or infection has been introduced and has meanwhile significantly broadened our understanding of the immune response [4–6]. Endogenous danger signals released from necrotic or stressed cells which trigger the inflammatory response after trauma have been termed alarmins or danger-associated molecular patterns (DAMPs) [6, 7]. Initially, it was believed that apoptotic cells are not a source for DAMPs, but it became meanwhile evident that DAMPs can also be released during a specific modality of programmed cell death, referred to as immunogenic apoptosis [8–10]. DAMPs share structural and functional similarities with exogenous, conserved microbial surface structures released from invading microorganisms, so-called pathogen-associated molecular patterns (PAMPs), which, like DAMPs, are recognized by a set of receptors, termed pathogen-recognition receptors (PRRs) [4, 11–15]. However, this definition of DAMPs is not always used consistently, and sometimes endogenous alarmins and exogenous PAMPs are collectively classified as danger-associated molecular patterns (DAMPs) [6]. 

Well-known alarmins include but are not limited to heat shock proteins (Hsp), hyaluronan, uric acid (UA, monosodium urate), galectins, thioredoxin, adenosine, high-mobility group box protein 1 (HMGB1), interleukin-1*α* (IL-1*α*), and interleukin-33 (IL-33) [6]. As unique features, HMGB1, IL-1*α*, and IL-33 exert dual functions as intracellular transcription factors and as extracellular inflammatory mediators. 

In this paper, we focus on the role of the dual function DAMPs in the initiation of the immune response after trauma. Moreover, we shed light on recently discovered mechanisms of activation of innate immunity by mitochondrial DAMPs released from disrupted cells which bear bacterial molecular motifs similar to PAMPs due to their endosymbiotic origin.

## 2. High-Mobility Group Box Protein 1

HMGB1 was originally described as a DNA-binding nuclear protein that acts as a transcription factor [16]. A decade ago, HMGB1 has been rediscovered as a proinflammatory cytokine in sepsis and endotoxemia which, under these conditions, is released downstream of the early cytokine production [17]. Meanwhile, HMGB1 has emerged as a prototypical DAMP and has been shown to play an important role in the response to sterile injury, such as hemorrhagic shock and ischemia/reperfusion injury [18]. As a ubiquitous protein, HMGB1 is virtually expressed by all cell types with a nucleus. 

HMGB1 can be released by active secretion predominantly from macrophages and monocytic cells but also from other cell types that are exposed to proinflammatory cytokines or bacterial products [19]. Besides PAMPs and cytokines, active HMGB1 secretion from monocytes can be triggered by the complement activation product C5a [20]. This mechanism not only seems to play a role in sepsis but also in sterile injury since in patients with severe trauma levels of HMGB1 correlate with the extent of complement activation [21]. Interestingly, active HMGB1 secretion is controlled by the autonomic nervous system as activation of the cholinergic anti-inflammatory pathway suppresses HMGB1 secretion from macrophages [22]. In this context, it has been postulated that the spleen is an abundant source for HMGB1 and that mediator secretion by splenic macrophages in the red pulp is under influence of the vagus nerve [23, 24]. 

In its role as an endogenous danger signal, passive release of HMGB1 from necrotic or disrupted cells stimulates innate immunity, while it was initially believed that HMGB1 is not released from apoptotic cells [16]. However, recent research revealed that after initial nuclear retention in apoptotic cells HMGB1 may be released during late apoptosis (secondary necrosis) due to increased cellular permeability and nucleosomal degradation [25]. 

When actively released by macrophages, HMGB1 undergoes posttranslational modifications, including acetylation, phosphorylation, and methylation [25–27]. Moreover, posttranslational redox modifications of certain cystein residues regulate the activity of (actively and passively released) HMGB1, including receptor interaction and subsequent signaling events [25, 28]. The redox sensitivity of HMGB1 has particular ramifications for systemic inflammation and sepsis with increased oxidative stress and release of reactive oxygen species (ROS). Oxidation of HMGB1 not only occurs in necrotic cells but also during apoptosis, which is associated with generation of ROS by mitochondria [29]. On the other hand, HMGB1 can promote the production of ROS in neutrophils [30]. It has been postulated that oxidation might temporarily inactivate HMGB1, and in turn, the activity of HMGB1 is prolonged and maintained in a reduced environment [29, 31].

With respect to its functional roles in inflammation, HMGB1 exerts pleiotropic proinflammatory effects on various organ systems. These effects include activation of phagocytic and endothelial cells and the loss of epithelial barrier functions, resulting in typical signs of inflammation and other symptoms, collectively referred to as “sickness syndrome” [32, 33]. In addition, HMGB1 promotes processes required for host defense, tissue repair, and regeneration, including chemotaxis, angiogenesis, maturation of dendritic cells, and recruitment and proliferation of stem cells [34]. Other reports suggest that HMGB1 amplifies the inflammatory response by binding endogenous and exogenous inflammatory mediators, such as cytokines or endotoxins [35, 36]. In fact, the intrinsic capacity of HMGB1 to trigger immune responses has been questioned lately since recombinant HMGB1 failed to induce cytokine production *in vitro*. A possible explanation for these conflicting results might be the redox state of cysteine residues since commercially available preparations of HMGB1 may contain reducing agents [25]. In the case of formation of HMGB1-containing immunostimulatory complexes, it has been suggested that inflammation is primarily promoted through the receptor of the partner molecule in independence of the redox state of HMGB1 [25, 37]. However, to date only limited information is available about the mechanisms, kinetics, and conformational changes involved in HMGB1-complex formation. 

The activities of HMGB1 are mediated through interaction with pathogen-recognition receptors, which also recognize products from invading microorganisms. HMGB1 has been shown to be a ligand of various toll-like receptors and to signal through TLR2 and TLR4, the latter being required for HMGB1-mediated activation of macrophages and the development of secondary damage in ischemia/reperfusion injury [18, 38–40]. In addition to TLRs, HMGB1 interacts with the receptor for advanced glycation endproducts (RAGE) on a variety of cell types which is involved in the initiation of a rapid and sustained inflammatory response as well as in mediating the chemotactic and mitogenic activities of HMGB1 [6, 41, 42].

However, RAGE not only senses and transmits danger upon HMGB1 release but also contributes to inactivation or neutralization of HMGB1 in form of soluble RAGE, which is released simultaneously to HMGB1 in severe trauma [43, 44]. But it is also conceivable that soluble RAGE functions as a carrier to convey HMGB1 activities to remote tissues/cells, as it has been suggested for various cytokines, although this putative mechanism of action currently lacks direct evidence. Binding of HMGB1 to thrombomodulin on endothelial cells represents another mechanism by which HMGB1-triggered inflammation can be attenuated [45]. On the other hand, interference of HMGB1 with the thrombin-thrombomodulin complex may inhibit the anti-inflammatory protein-C pathway. Furthermore, HMGB1 can drive coagulation towards a procoagulatory state by stimulating tissue factor expression and inhibiting tissue plasminogen activator on endothelial cells, putatively paving the way for the development of manifest coagulopathy and disseminated intravascular coagulation [46].

The knowledge about HMGB1 as an inflammatory mediator gained during the last decade after its rediscovery is mainly based on results from experimental studies, with models of sepsis and endotoxemia in particular. In the setting of experimental sepsis, HMGB1 has been defined as a late mediator, as compared to other cytokines, such as IL-6 and TNF-*α* [17]. Antibody-induced neutralization or use of recombinant A box domain of HMGB1 (antagonist of B box proinflammatory activity) in sepsis could improve the outcome, even when applied after the onset of disease [17, 47, 48]. Other preclinical models, in which blockade of HMGB1 led to promising results, include arthritis, ischemic brain injury, liver injury, and organ transplantation [25]. In severe trauma in humans, HMGB1 acts as an early proinflammatory mediator, which is systemically released within 30 to 60 minutes, peaking from 2 to 6 hours after injury [21, 49]. The concentration of HMGB1 correlates with the severity of injury and the systemic inflammatory response. Moreover, patients who develop organ dysfunction and nonsurvivors of severe trauma show higher levels of HMGB1 [21]. In a conflicting report, no correlation between increased HMGB1 levels after trauma and injury severity or parameters of patient outcome was found, which might be due to a rather small sample size in the latter study [49]. However, there are striking differences in the absolute values of HMGB1 concentrations measured in the patient populations of both studies, which may reflect the difficulty in HMGB detection. As a matter of fact, current standard detection assays do not distinguish between different forms of HMGB1 as a result of posttranslational changes, redox reactions, or complex formation [25]. In contrast to sterile injury, the peak of HMGB1 release during sepsis occurs during later stages of the disease, and the levels of HMGB1 do not always decrease in patients who have recovered from sepsis [17, 50]. Although neutralization of HMGB1 has been protective against tissue injury in numerous preclinical models of inflammatory diseases, the complexity of its mechanisms of action currently precludes the clinical use of HMGB1-neutralizing agents, and clinical studies targeting HMGB1 have not been performed at present.

Owing to its pleiotropic proinflammatory activities, HMGB1 still represents a promising therapeutic target in various inflammatory conditions. However, targeting HMGB1 for protection in sterile injury and infection-associated inflammation in the clinical setting requires thorough understanding of the underlying molecular mechanisms. 

## 3. Interleukin-1*α*

The interleukin 1 family (IL-1F) currently consists of 11 known members [51]. Their effects in inflammation are complex as they have anti-inflammatory as well as proinflammatory properties. In general, IL-1 activates lymphocytes, enhances the defensive activity of monocytes and macrophages by inducing the production of inflammatory mediators, and acts as costimulant on natural killer cells [52]. IL-1 is divided into 2 subtypes, IL-1*α* and IL-1*β*. IL-1*β* is the most thoroughly investigated member of the IL-1F due to its role in autoimmune diseases [53]. Interleukin-1 receptor antagonist (IL-1Ra) is a specific inhibitor of IL-1*α* and IL-1*β* on their membrane-bound receptor IL-1R, and generic IL-1Ra is routinely used in the treatment of patients with rheumatoid arthritis [54].

IL-1*α* belongs to the group of dual function alarmins (formerly also known as endokines), describing the ability to induce an inflammatory response upon release by necrotic cells besides intracellular functions in intact cells [7]. In contrast to IL-1*β*, IL-1*α* is constitutively expressed in epithelial cells, keratinocytes, and fibroblasts. Its precursor molecule (pIL-1*α*) is also biologically active. 

Cells constitutively expressing IL-1*α* rarely secret it in an active manner. From these cells, IL-1*α* is only released after loss of cellular integrity. In contrast, monocytes and macrophages do not constitutively express IL-1*α* but are capable of de novo synthesizing IL-1*α*. Upon activation of monocytic cells, membrane calpain is activated to cleave pIL-1*α*, followed by secretion of IL-1*α*. In contrast to cellular necrosis, IL-1*α* remains attached to chromatin during apoptosis, which reduces its intracellular mobility and possibly limits its passive release [55]. Thus, IL-1*α* is predominantly released by necrotic cell disintegration, but stays intracellular under physiological conditions, during apoptosis, and even in the presence of inflammatory diseases [56–59]. As mentioned above, the precursor can be cleaved by membrane-bound calpain, a calcium-activated cysteine protease, which is not found in all cell types expressing pIL-1*α* [60]. Both the uncleaved (pIL-1*α*) and the mature form of IL-1*α* can bind to the IL-1R, but membrane-bound IL-1*α* can also exert juxtacrine functions in a receptor-independent manner [61]. Like IL-33 (see below) but unlike other members of the IL-1F, IL-1*α* not only acts on intra- or extracellular membrane receptors but also as a nuclear transcription factor [62]. While pIL-1*α* contains the sequence for the nuclear localization site, the mature form (IL-1*α*) has no ability to function as a transcription factor [63]. In cultured resting cells, pIL-1*α* is distributed evenly in the cytoplasm. After exposition to inflammatory stimuli, such as lipopolysaccharides (LPS) or TNF*α*, pIL-1*α* locates to the nucleus without further processing and acts as a transcription factor. This leads to the IL-1R-independent production of NF-*κ*B and proinflammatory cytokines, such as IL-6 and IL-8 [63]. However, the biological role of constitutively expressed IL-1*α* is not entirely clear. In unstimulated cells expressing IL-1*α*, large amounts of intracellular IL-1Ra are present at the same time, competing for binding sites [64]. IL-1*α* has a costimulatory effect on T-cell function and is expressed consistently by thymic epithelial cells, but mice deficient of IL-1*α* show normal antibody production and proliferation [51, 65]. The expression of interferon-*γ* largely depends on IL-1*α* and antibodies to IL-1*α* but not IL-1*β* block its activity [66]. Antibodies to IL-1*α* inhibited the immune response in sterile inflammation in mice, corroborating its role as an alarmin [67]. Interestingly, mesothelial cells have been proposed to play a key role in sensing cell death. Exposed to recombinant IL-1*α*, they produce CXCL-1, a cytokine with neutrophil attractant properties [68, 69]. The intraperitoneal injection of lysed cells *in vivo* leads to neutrophil infiltration that is markedly reduced in CXCR2- (receptor to CXCL-1-) deficient mice. The CXCL-1 production following exposure to cytosolic extracts of necrotic tissues can be abolished by IL-1Ra or in IL-1*α*-deficient in mice. Conversely, injection of lysed CXCR2^−/−^ cells results in reduced neutrophil recruitment as compared to cell lysates from wild-type mice [69]. However, mesothelial bone marrow-derived cells cannot only sense IL-1*α* but also secrete it upon exposure to lysed cells *in vitro* [70].

Although IL-1*α* is known for a fairly long time, the information about its distinct roles in the *in vivo* setting in SIRS is limited since most clinical studies focused on the role of IL-1*β* rather than the other subtype IL-1*α*. Moreover, only few studies have investigated the isolated effects of either pIL-*α* or mature IL-*α* [63]. With respect to trauma, there is only a single report available investigating the release of IL-1*α* in patients with systemic inflammation after accidental injury. However, in this study IL-1*α* was not detectable in any patient at any time point investigated over a 5-day period [71]. While this does not necessarily mean that IL-1*α* is not systemically released during trauma, the findings might be based on limitations of the detection assays used two decades ago. Other possible explanations include unknown internal clearance mechanisms of systemically released IL-1*α*, complex formation with endogenous antagonists or other inflammatory mediators, or tissue sequestration. In humans, injection of IL-1 leads to symptoms of systemic inflammation, including fever, myalgia, arthralgia, and a lowered pain threshold. Although preclinical studies treating SIRS and sepsis in mice with IL-1Ra revealed promising results, it failed to significantly reduce the overall mortality of sepsis in humans in large double-blind, placebo-controlled clinical trials [72]. To our knowledge, no clinical studies have been conducted yet to test the effect of specific blockade of IL-1*α* in systemic inflammation after trauma in humans. 

In summary, IL-1*α* fulfills the definition criteria for a dual function alarmin since it not only functions as a powerful inductor of systemic inflammation when released into the extracellular space but also acts as a nuclear transcription factor. However, although IL-1*α* represents the first dual function protein described, its particular role in sterile inflammation and trauma is less thoroughly investigated than HMGB1, its archetypical partner in crime. 

## 4. Interleukin-33

Due to similarities to IL-1*α* and HMGB1 with respect to constitutive, nuclear tissue expression and passive release after loss of cellular integrity, it has been suggested that IL-33 represents the third dual function protein of the alarmin family [73]. IL-33 was previously known as nuclear factor from high endothelial venules (NF-HEV) and was identified by computational data base analysis as the ligand for ST2 (also known as IL-1RL1), which, until then, was designated as an orphan receptor [74]. IL-33, the latest member of the IL-1 cytokine family, is mainly expressed in structural and lining cells, including endothelial cells, fibroblastic reticular cells of lymphoid tissues, and epithelial cells of tissues exposed to the environment [73]. In the absence of inflammatory stimuli, IL-33 localizes to the nucleus, which is mediated by the amino terminus of full-length IL-33 [75]. 

IL-33 was originally considered to be actively released to the extracellular space after proteolytic cleavage of its precursor pIL-33 [74]. The active release of IL-33 from macrophages can be triggered by PAMPs, such as LPS, while dendritic cells or mast cells have not been found to be source of active IL-33 secretion [76]. In contrast to other members of the IL-1 family, active IL-33 secretion is independent of caspase-1 and caspase-8 (required for cleavage of pIL-1*β* and/or pIL-18) or calpain (required for cleavage of pIL-1*α*) [76]. Although recombinant pIL-33 is cleaved by recombinant caspase-1 *in vitro* [74], the *in vivo* role of caspase-1 in the cleavage of pIL-33 (full-length IL-33) remains controversial [76].

With respect to its role as a DAMP, it has been demonstrated that nuclear, full-length IL-33 is biologically active and can be released following cellular damage [77–79]. It has been suggested that different biologically active forms of IL-33 exist [80, 81]. However, to date the distinct roles of various forms of IL-33 as well as the corresponding mechanisms of release are enigmatic, as are potential posttranslational modifications or environment-dependent functional/conformational alterations of IL-33. Although our knowledge about IL-33 secretion is limited due to its recent discovery, passive release of IL-33 from necrotic tissues seems to be the major pathway since pIL-33 does not exhibit typical peptide sequences for active secretion, and full-length IL-33 is thought to be the biologically most active form [78]. In line with this, inactivation of IL-33 through proteolytic cleavage during apoptosis may limit the release of biologically active full-length IL-33. 

With respect to the mechanisms of release and activation/inactivation of IL-33, various studies reported conflicting results. Initially, it was believed that IL-33 is activated through caspase-1-dependent cleavage of pIL-33 into an active form [74]. However, more recently, it has been demonstrated that the functional activity of IL-33 is independent of caspase-1-cleavage and that IL-33 may even be inactivated by caspase-1 [77, 80]. According to another study, cleavage of IL-33 into less active forms is presumably mediated by the proapoptotic caspases-3 and -7, while caspase-1 cleavage only seems to play a minor role under physiological conditions [78]. Moreover, an alternative splice variant of IL-33 has recently been identified, the functional role of which has yet to be defined [81]. 

As a dual function protein, IL-33 is active as a nuclear transcription factor and as a cytokine. But unlike IL-1*α* and HMGB1, IL-33 exerts repressive transcriptional activity and features some anti-inflammatory properties [82]. IL-33 can activate cells of the innate as well as the adaptive immune system via interaction with membrane ST2, which, in particular, is abundantly expressed on the surface of T helper 2 (Th2) cells and mast cells [83]. Through interaction with membrane-bound ST2, IL-33 promotes Th2-type immune responses, with enhanced production of the anti-inflammatory cytokines IL-5 and IL-13, drives the differentiation of naïve T cells towards a Th2 phenotype, and functions as chemotactic factor in Th2 cell mobilization [84, 85]. On mast cells, IL-33 triggers the production and release of proinflammatory cytokines and chemokines, such as IL-1*β*, IL-6, or TNF*α*, promotes maturation, and induces degranulation [79, 86, 87]. Therefore, IL-33 has been attributed to mediate anaphylactic shock [88]. Furthermore, IL-33 amplifies the polarization of alternatively activated macrophages, upregulates TLR4, and enhances TLR4-mediated cytokine production by macrophages [79, 89, 90]. The receptor for IL-33, ST2, exists in different splice variants, resulting in a localized form bound to the cellular membrane and a soluble form [91]. The soluble variant, termed sST2, is generated by alternative splicing and is not thought to induce signaling, therefore acting as a decoy receptor for IL-33 [79]. Similar to IL-33, sST2 has been linked to the pathogenesis of various inflammatory conditions, including sepsis, asthma, autoimmune diseases, and cardiovascular diseases [92–95]. In general, sST2 is considered as a marker of poor prognosis.

Since IL-33 represents the most recent member of dual-function DAMPs, to date there are no specific clinical data available on the role and kinetics in patients with severe trauma. However, there is indirect evidence of its involvement in trauma since it has been demonstrated that patients with SIRS after major trauma or sepsis exhibit elevated levels of the soluble receptor sST2, possibly associated with a poor outcome [10]. In contrast to sterile injury and trauma, the role of IL-33 in sepsis is better defined. In experimental sepsis, IL-33 has beneficial effects by enhancing the accumulation of neutrophils at the site of infection and reducing systemic but not local proinflammatory responses, resulting in an improved outcome [96]. However, it remains to be determined in future studies if administration of IL-33 in fact represents a therapeutic strategy in the clinical treatment of patients with sepsis or SIRS. 

Taken together, the novel cytokine and alarmin IL-33 functions as an important activator of the innate and the adaptive immune system. However, its particular roles remain enigmatic to date since, depending on the environment, IL-33 can either play a beneficial role and lead to the resolution of inflammatory processes, or, on the other hand, IL-33 can contribute to aggravation of inflammation.

## 5. Mitochondrial DAMPs

Bacterial infection and major trauma both can elicit responses that are summarized as systemic inflammatory response syndrome (SIRS) or sepsis, respectively [97–99]. The clinically similar phenotype led to the hypothesis that the molecular pathways may have resemblances as well. According to the “danger theory,” traumatic cell destruction causes release of substances that are usually hidden intracellularly, but signify “danger” to the host once they appear in the extracellular environment [5]. Searching for endogenous agents that provoke activation of the immune response, mitochondria could be recently identified as potent effectors. Based on striking similarities between bacteria and mitochondria, the endosymbiotic theory was established already more than a century ago, according to which bacteria with the ability to conduct respiration were incorporated by eukaryotic cells by endocytosis [100]. However, a direct link between the development of SIRS and the release of mitochondrial constituents following cellular damage in trauma could not be established until recently when it has been shown that mitochondrial DAMPs (MTDs) are markedly elevated in severely injured patients [101]. These MTDs mainly comprise circular DNA strands containing CpG DNA repeats and N-formylated peptides [102]. 

Mitochondrial DNA (mtDNA) is released by shock and can directly activate neutrophils after binding to TLR9 [103, 104]. Moreover, proinflammatory cytokine secretion by monocytes/macrophages was found to be augmented after exposure to mtDNA [105]. Mitochondrial DNA released after trauma can directly activate neutrophils after binding to TLR9 and activation of the intracellular p38 MAPK signaling pathway [103, 104]. In accord, systemic administration of mtDNA in mice resulted in systemic inflammation and the development of lung injury [101]. Therefore, it has been hypothesized that the release of soluble mitochondrial degradation products may be the missing link between tissue injury and sterile SIRS [106]. As an example for the relevance in the clinical setting, it has been shown that femoral reaming during fracture fixation causes release of MTDs into the wound and circulation, which may be associated with the development of acute lung injury [107].

N-formyl peptides synthesized by mitochondria are strong chemoattractants as they closely resemble those derived from bacteria [108]. They bind to formyl-peptide receptors (FPRs) and its functional variant FPR-like 1 receptor (FPRL1) [109]. It has been demonstrated that isolated mitochondrial peptide fragments bind to FPRL1 and trigger proinflammatory responses through chemotactic effects [110]. Especially phagocytic cells, which are specialized in defending the host against invading microorganisms, express FPR and FPRL1 on their surface [111]. Signal transduction through G-proteins following engagement of FPR and FPRL1 results in chemotaxis, Ca^2+^ mobilization, activation of MAP-kinase signaling pathways, cytokine production and release, desensitization of other chemoattractant receptors, and respiratory burst [112–115]. Interestingly, isolated N-formyl peptides do not trigger an inflammatory response in monocytes unless coupled with mitochondrial transcription factor A (TFAM), a homologue to the nuclear HMGB1 [116]. These effects of mitochondrial N-formyl peptides can be attenuated by receptor antagonists or silencing of FPR [116]. 

With respect to the role in ATP-generation, mitochondrial function was found to be reduced in sepsis and trauma patients, resulting in ATP-depletion and eventually cellular necrosis [117]. Furthermore, the mitochondrial production of ROS is enhanced during the inflammatory response in trauma and sepsis. It has been suggested that mitochondrial ROS can regulate NF-*κ*B levels in immune cells, thereby inducing an inflammatory response [117]. 

Moreover, mitochondria have emerged as crucial mediators in the induction of apoptosis during SIRS in trauma and shock [117, 118]. Besides this well-known form of programmed cell death, there is now evidence that necrosis can also occur in an organized manner, called necroptosis. It can be induced by so-called death receptors, such as TNF-R, during SIRS and involves activity of the receptor-interacting protein kinase pathway (RIPK). Activation of RIPK1 and RIPK3, a signaling complex also referred to as necrosome, is followed by active disintegration of mitochondrial and plasma membranes [119, 120]. In a recent report, RIPK3-deficient mice were protected from lethality in experimental models of sterile SIRS and polymicrobial sepsis [119]. Interestingly, levels of mitochondrial DNA in plasma were lower in RIPK3-deficient mice, suggesting reduced tissue damage in absence of RIPK3 [119].

In summary, besides their known functions in ATP-generation, apoptosis, biosynthesis, and calcium homeostasis, mitochondria play an important role in activating innate immunity since they contain constituents of their bacterial ancestors which are potentially immunogenic [121]. 

## 6. Conclusions

The dual-function alarmins HMGB1, IL-1*α*, and IL-33 represent crucial mediators in the initiation and perpetuation of the inflammatory response following loss of cellular integrity. While HMGB1, IL-1*α*, and IL-33 share the unique features of acting as transcription factors and extracellular mediators of inflammation, each dual-function protein exerts distinct functions, which we are just beginning to understand. Further, the dual-function mediators substantially differ in their mechanism of action and release. Based on the information available to date, the role of the dual-function mediators in systemic inflammation provides a possible explanation for the enigmatic question of why patients with severe (sterile) injury present with a syndrome that is indistinguishable from sepsis. The discovery of mitochondrial DAMPs, which activate the immune response after cellular disruption by mimicking bacterial infection, has opened up a new avenue for the investigation of danger sensing and transmission. However, future basic science research as well as clinical studies in this fascinating field are necessary to further unravel the complexity of the host response after trauma and tissue damage. In the setting of sterile injury and trauma, the roles of IL-1*α* and IL-33 and their various forms as a result of posttranslational modifications and corresponding environments need to be defined in detail. In this context, novel preclinical models of trauma may help characterize the role of DAMPs and investigate mechanisms/kinetics of release after tissue injury in single-organ injury and multi-system trauma, followed by rapid transfer of findings to the setting of human disease. Further possible future research directions may include posttranslational modifications of DAMPs and their dynamics after tissue injury, which may be associated with alterations of the functional roles, ranging from activation of inflammation to tissue repair, or even anti-inflammatory activity. In addition, only little is known about mutual interactions of DAMPs prior and after passive or active release and direct crosstalk with other mediators of inflammation and signaling systems. Moreover, mechanisms of intracellular retain of DAMPs during different modalities of cell death appear to be an interesting field for future research. With respect to potential therapeutic strategies, besides agents that neutralize or block DAMPs, the development of compounds that cause intracellular DAMP retention and limit DAMP release upon tissue damage might represent a promising approach. The versatility of DAMPs and associated signaling systems is an impressive example for the plasticity of innate immunity, and with increasing understanding of the underlying mechanisms and interactions, routine clinical application of DAMP-targeting strategies for the treatment of patients with SIRS may be in reach.
